# Caregiver Feeding Practices and Their Association With Nutritional Status in Children Aged 0-5 Years: A Descriptive Cross-Sectional Study From a Tertiary Care Center in North India

**DOI:** 10.7759/cureus.108756

**Published:** 2026-05-12

**Authors:** Sharon R John, Ruby Singh

**Affiliations:** 1 Pediatrics and Child Health, Christian Medical College and Hospital, Ludhiana, Ludhiana, IND

**Keywords:** anthropometry, caregivers' influence, children, feeding pattern, malnutrition

## Abstract

Background

Adequate nutrition in the first five years of life is critical for optimal physical growth, cognitive development, and long-term health outcomes. Caregiver feeding practices are an important factor associated with nutritional outcomes in young children with a spectrum of behaviors ranging from responsive feeding to more controlling strategies. While responsive feeding has been linked to better growth outcomes, the impact of specific feeding behaviors on child nutritional status in tertiary pediatric settings in North India remains poorly characterized. Therefore, this study aimed to evaluate caregiver feeding practices using a validated tool and examine their association with nutritional status in children aged 0-5 years.

Objectives

The objective of this study is to assess caregiver feeding practices using the Feeding Practices and Structure Questionnaire (FPSQ) and examine their association with nutritional status (Indian Academy of Pediatrics {IAP} grading) in children aged 0-5 years attending the outpatient department of a tertiary care pediatric center, in order to identify feeding behaviors associated with malnutrition in this setting.

Materials and methods

A descriptive cross-sectional study was carried out over a period of three months in the outpatient department of pediatrics at Christian Medical College, Ludhiana. A total of 150 caregivers of children aged 0-5 years were enrolled. After obtaining informed consent, each caregiver was administered a structured questionnaire (Feeding Practices and Structure Questionnaire {FPSQ}), which is a validated tool assessing both feeding practices and feeding patterns. Simultaneously, anthropometric measurements of the children were recorded, and their nutritional status was classified using the Indian Academy of Pediatrics (IAP) grading system. The collected data were analyzed to assess the association between feeding behavior and nutritional status.

Results

Out of 150 children, 83.3% were normally nourished, 16% had mild malnutrition, and 0.7% had moderate malnutrition. Responsive feeding behaviors such as feeding on demand and shared family meals were the most commonly reported practices. In the milk-feeding group, feeding on demand showed a significant association with better nutritional status (p = 0.015). In the semi-solid group, parent-led feeding was significantly associated with malnutrition (p = 0.008).

Conclusion

Feeding on demand was associated with better nutritional outcomes in milk-fed children, while parent-led feeding was associated with malnutrition in the semi-solid-feeding group. These findings are hypothesis-generating, given the cross-sectional design, and reverse causality cannot be excluded. Responsive feeding counseling may be a relevant avenue for nutritional improvement in routine pediatric outpatient care.

## Introduction

Early life nutrition forms the cornerstone of healthy growth and cognitive development, with infant and young child feeding (IYCF) practices being pivotal in determining long-term health trajectories [1]. Parental feeding practices encompass the goal-oriented strategies caregivers employ when feeding their children, including restricting certain foods, pressuring children to eat, monitoring dietary intake, and modelling healthy eating behaviors [2]. Beyond physical growth, early nutritional experiences also shape food preferences, dietary habits, and attitudes toward eating that may influence lifestyle-related disease risk well into adulthood, making young children and their caregivers key targets for preventive health interventions. Among the factors shaping child dietary outcomes, caregiver nutritional knowledge stands out as one of the most amenable to change through targeted intervention [3].

Children's appetitive traits have been shown to influence their nutritional status. Those exhibiting lower appetite-related behaviors, such as slower eating pace, greater satiety responsiveness, food fussiness, and emotional undereating, tend to have lower body fat levels [4]. Parental dietary habits, feeding approaches, and modelled behaviors collectively shape a child's food environment and dietary intake [5]. Caregivers of preschool children have also been observed to adjust their feeding strategies based on the child's weight and eating behavior by increasing pressure to eat when a child shows poor food interest while imposing greater dietary restrictions in children who are highly food-responsive [6].

Despite progress across several health indicators, India continues to face a significant dual burden of undernutrition alongside rising rates of overweight and obesity, both of which disproportionately affect vulnerable populations [7]. While India faces this dual burden of undernutrition and overweight, the present study focuses specifically on undernutrition in young children attending a tertiary care pediatric outpatient setting, as this remains the primary nutritional concern in this population. Malnutrition remains one of the primary contributors to under-five mortality across low- and middle-income countries, making it one of the most critical unresolved challenges in global pediatric health [8]. When caregivers have adequate knowledge about nutrition, their feeding choices tend to be more appropriate. Some studies report significant relationships with specific anthropometric indicators, such as wasting, but not with overall malnutrition [9].

Feeding difficulties in early childhood are common and, when not identified and managed early, can have lasting consequences, including impaired development and an increased risk of disordered eating patterns in later years [10]. Food consumption behavior is inherently complex, and focusing on individual nutrients or food items fails to capture the multidimensional nature of dietary patterns. A comprehensive understanding of the factors influencing children's eating behaviors is therefore essential [11].

The Feeding Practices and Structure Questionnaire (FPSQ), developed by Jansen et al., is a theoretically informed and well-validated instrument designed to capture the full range of caregiver feeding behaviors from infancy through the toddler years [12]. Although the link between feeding practices and child nutrition is increasingly recognized, studies from tertiary pediatric outpatient settings in North India using validated tools remain scarce. This study aimed to assess caregiver feeding practices using the FPSQ and to examine their association with the nutritional status of children between zero and five years of age seen at the department of pediatrics at Christian Medical College, Ludhiana.

## Materials and methods

The descriptive cross-sectional study was carried out in the outpatient department of the department of pediatrics at Christian Medical College, Ludhiana. Prior to initiation, ethical clearance was obtained from the Institutional Ethics Committee of Christian Medical College, Ludhiana (approval number: IECBMHR/202411-506/Apprvl-STS-Proj/CMC&H).

The study population included caregivers of children aged 0-5 years attending the pediatric outpatient department, and also, the primary feeding person for the child was enrolled. Caregivers of children with dietary restrictions, physician-advised dietary modifications (e.g., celiac disease, nephrotic syndrome, and diabetes mellitus), or neurological sequelae were excluded. The objectives of the study were discussed in detail with the caregivers, and they were provided a participant information sheet, following which written informed consent was obtained.

First, the demographic data of the caregiver and the child were obtained and recorded, along with the socioeconomic status (SES), which was measured by the Modified Kuppuswamy Scale, a widely validated tool used in Indian research settings [13]. The anthropometric measurements, such as the weight and height/length (using a stadiometer/infantometer) of the children included in the study, were recorded. Weight was measured using the pediatric weighing scale (Avery, Smethwick, United Kingdom) present in the outpatient department. Length was measured using an infantometer/stadiometer (length was recorded for children less than two years and height from more than two years of age), and the grade of malnutrition using the Indian Academy of Pediatrics (IAP) grading was calculated based on the anthropometric measurements.

A structured questionnaire was administered to 150 caregivers of children between the ages of zero and five years. Feeding practices were assessed using the Feeding Practices and Structure Questionnaire (FPSQ) for infants and toddlers developed by Jansen et al. [12]. The FPSQ is published under a Creative Commons Attribution 4.0 International License (CC BY 4.0) and was used without modification in accordance with its terms.

The questionnaire was administered in the participants' preferred language. For participants unable to read, the questions were explained verbally to ensure understanding. The tool underwent pilot testing prior to the study, and content validity was assessed through review by subject experts. Formal forward-backward translation and linguistic validation of the questionnaire were not performed. It consisted of two versions: the milk-feeding version and the solid-feeding version. The milk-feeding version was administered to caregivers of children who are primarily fed milk. The solid-feeding version was administered to parents who had initiated complementary diets for their children, along with milk. The milk-feeding version covered four domains, which were demand versus routine feeding, parent-led feeding, persuasive feeding, and the use of food to calm, comprising 18 items in total. The solid-feeding version retained these four domains across 21 items and additionally included two subscales relevant from 12 months onward: family meal environment and the use of non-food rewards, assessed with 13 further items.

Scoring

Each subscale contained between four and seven items rated on a five-point Likert scale (1 = never and 5 = always), with designated items reverse-scored. The single continuous scores were computed as the mean (M) of constituent item scores, such that higher values reflected the greater frequency of that feeding practice (e.g., mean score for parent-led feeding = PLF1 + PLF2 + PLF3 + PLF4 + PLF5 + PLF6 / 6). These scores were correlated with the anthropometric measurements taken and the relationship between feeding patterns and anthropometric measures, highlighting that any significant associations were summarized.

Sample size

The sample size was calculated based on national estimates of undernutrition among under-five children in India, reported at approximately 35.7% according to the National Family Health Survey-5 (NFHS-5) [14]. Using a confidence level of 95% and an absolute precision of 8% and accounting for a 10% nonresponse rate, a minimum sample size of 138 participants was estimated. A total of 150 caregivers were enrolled to ensure adequate statistical power.

Statistical analysis

Statistical analysis was performed by using the Statistical Package for the Social Sciences (SPSS) software version 29.0 (IBM Corp., Armonk, NY). The data were collected, coded, categorized, and recorded in a Microsoft Excel sheet (Microsoft Corp., Redmond, WA). The categorical variables were represented as frequency (percentage), and continuous data were represented as mean ± standard deviation (SD). Group comparisons were performed using chi-square tests for individual feeding practice domains. Nutritional grade comparisons were restricted to well-nourished (Grade 0) and mildly malnourished (Grade 1) subgroups. The single case of moderate malnutrition (Grade 2) was reported descriptively only, as the absence of variance in a group of one individual precludes valid inferential analysis. The p-value of <0.05 was considered statistically significant. No correction for multiple comparisons was applied; results should therefore be interpreted with caution, given the potential for type I error.

## Results

Sociodemographic profile of caregivers and children

The sociodemographic profile of caregivers and children enrolled in the study is presented in Table 1. The mean age of caregivers was 29.93 ± 2.81 years (range: 23-36 years). The majority of caregivers were mothers (99.3%), with only one father participating. Most caregivers were well-educated, with 45.3% being graduates and 6.7% postgraduates, while smaller proportions had completed Grade 12 (23.3%) and Grade 10 (22.0%), and only 2.7% were illiterate. In terms of socioeconomic status, a majority belonged to the upper middle class (65.3%), followed by lower middle (18.7%) and upper class (12.7%), with very few participants from upper lower (2.7%) and lower (0.7%) classes. The mean age of the children was 1.85 ± 1.34 years (range: one month to five years), with a predominance of male children (62%). Most children were first-borns (56%), followed by second-borns (42%), while only 2% were of third or higher birth order. The mean weight and height/length of the children were 10.15 ± 3.27 kg and 80.2 ± 14.12 cm, respectively.

**Table 1:** Sociodemographic Characteristics of Caregivers and Children SD: standard deviation

Variable	Category	Frequency (n)	Percentage (%)
Caregiver age (years)	Mean ± SD	29.93 ± 2.81	Range: 23-36
Caregiver gender	Female (mother)	149	99.3
	Male (father)	1	0.7
Educational qualification	Illiterate	4	2.7
	Grade 10	33	22.0
	Grade 12	35	23.3
	Graduate	68	45.3
	Postgraduate	10	6.7
Socioeconomic status	Upper	19	12.7
	Upper middle	98	65.3
	Lower middle	28	18.7
	Upper lower	4	2.7
	Lower	1	0.7
Child age (years)	Mean ± SD	1.85 ± 1.34	Range: 0.08-5.0
Child gender	Male	93	62.0
	Female	57	38.0
Birth order	First	84	56.0
	Second	63	42.0
	Third or more	3	2.0
Child weight (kg)	Mean ± SD	10.15 ± 3.27	Range: 3.0-19.0
Child height/length (cm)	Mean ± SD	80.2 ± 14.12	Range: 50-106

Degree of malnutrition

**Figure 1:**
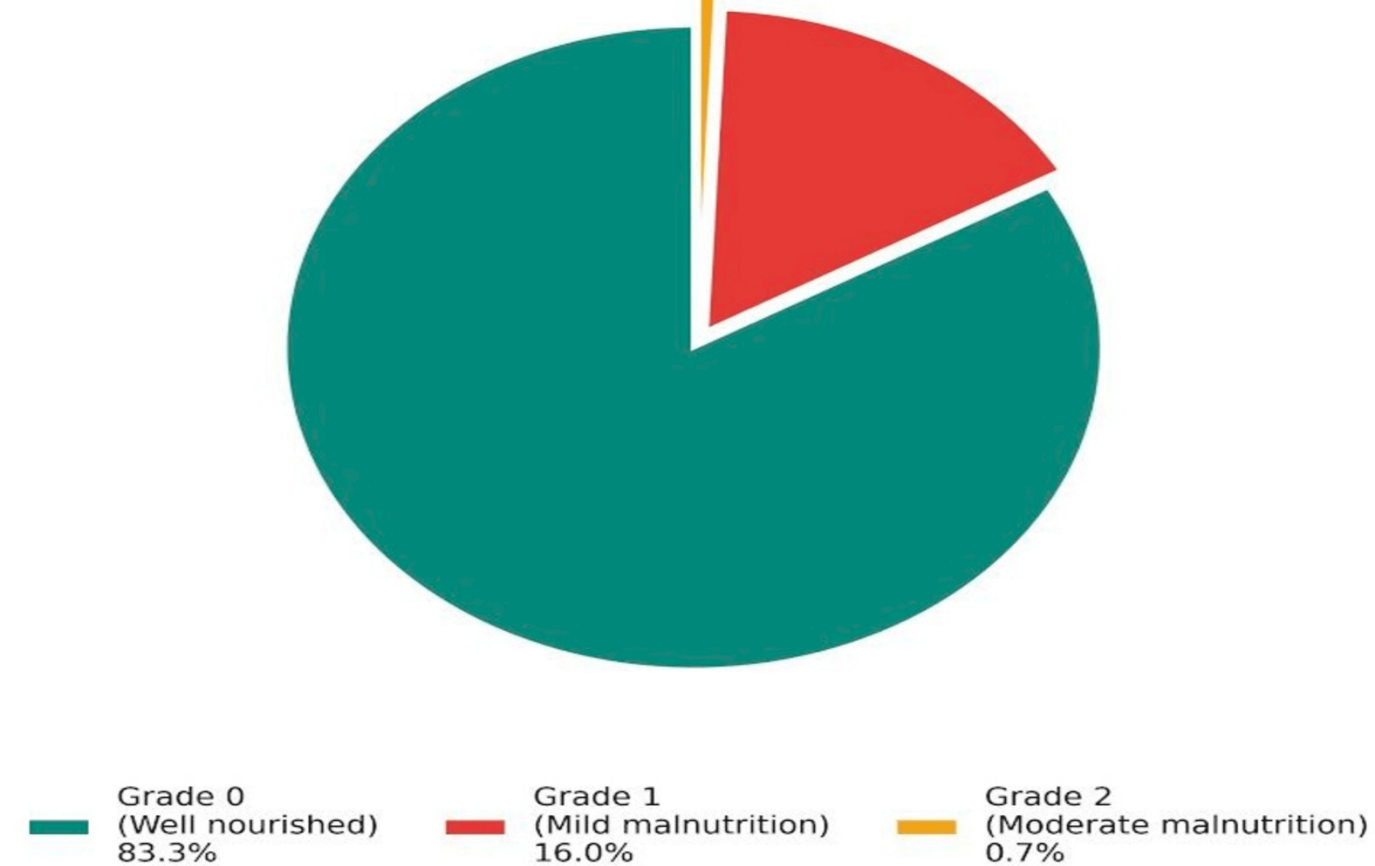
Degree of Malnutrition in Children Participating in the Study Distribution of malnutrition grades among children aged 0-5 years (N = 150) as classified by the Indian Academy of Pediatrics (IAP) grading system based on weight-for-age. Grade 0 = normal (≥80% of expected weight-for-age); Grade 1 = mild malnutrition (71%-80%); Grade 2 = moderate malnutrition (61%-70%). No cases of severe malnutrition (Grade 3 or 4) were identified in this study population

Based on anthropometric assessment and IAP classification of malnutrition, the majority of children in the study were classified as well-nourished (Grade 0), accounting for 83.3% (n = 125). Mild malnutrition (Grade 1) was observed in 16.0% (n = 24), while only one child (0.7%) had moderate malnutrition (Grade 2), as seen in Figure 1.

Version of the questionnaire

To assess caregiver feeding practices, two versions of the Feeding Practices and Structure Questionnaire (FPSQ) were used based on the child's current feeding stage. The semi-solid version was administered to the majority of caregivers (82.7%, n = 124), while the milk-feeding version was used for 17.3% (n = 26) whose children had not yet transitioned to semi-solid foods.

Feeding practices in the milk-feeding group

**Figure 2:**
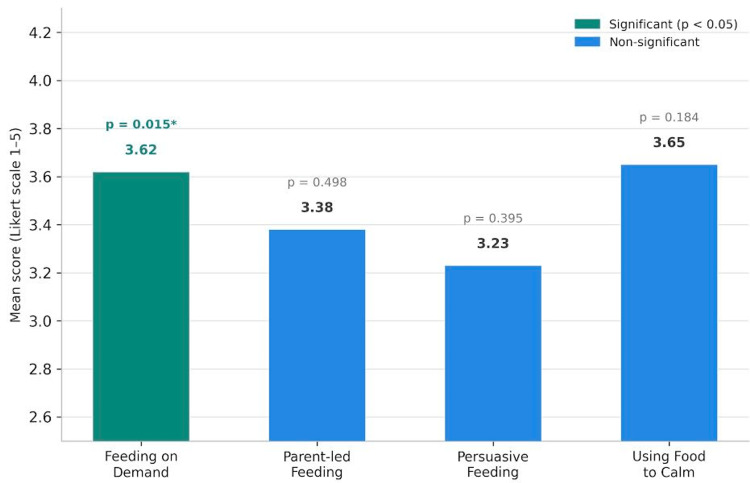
Graphical Representation of Mean Scores and Feeding Practices in Milk-Feeding Group Bar chart showing mean Feeding Practices and Structure Questionnaire (FPSQ) domain scores in the milk-feeding group (n = 26). Scores are plotted on a five-point Likert scale (1 = never to 5 = always). Higher scores indicate greater frequency of the respective feeding practice. Using food to calm showed the highest mean score (M = 3.65) and persuasive feeding the lowest (M = 3.23) *P < 0.05, statistically significant

Among the 26 caregivers who completed the milk-feeding version of the FPSQ, mean scores across the four domains revealed a preference for responsive feeding behaviors, as seen in Table 2. Scores are expressed as a mean on a five-point Likert scale (1 = never; 5 = always). Most common response refers to the modal Likert score. Interpretation indicates the qualitative label for the modal response. The highest scoring domain was using food to calm (M = 3.65; SD = 0.49), followed closely by feeding on demand (M = 3.62; SD = 0.57). Lower scores were observed in parent-led feeding (M = 3.38; SD = 0.70) and the lowest in persuasive feeding (M = 3.23; SD = 0.59) (Figure 2).

**Table 2:** Mean Scores of Feeding Practice in the Milk-Feeding Group P-values represent the association between each feeding practice and malnutrition grade assessed using the chi-square test for individual domains. The single case of moderate malnutrition (Grade 2) was excluded from all inferential comparisons. A p-value of <0.05 was considered statistically significant for chi-square analyses

Feeding practices	Mean scores	Most common response	Interpretation	P-value (feeding practice and malnutrition)
Feeding on demand	3.62	4	Often	0.015
Parent-led feeding	3.38	3	Sometimes	0.498
Persuasive feeding	3.23	3	Sometimes	0.395
Using food to calm	3.65	4	Often	0.184

Statistical associations between malnutrition and feeding patterns in the milk-feeding group

Descriptively, well-nourished children (Grade 0, n = 21) reported a higher mean total feeding score (M = 3.54; SD = 0.29) compared to mildly malnourished children (Grade 1, n = 5) (M = 3.20; SD = 0.33). These findings are presented as descriptive observations only. The formal inferential testing of total feeding scores was not performed, as the subgroup sizes, particularly the single case of moderate malnutrition (Grade 2), did not meet the minimum requirements for valid parametric comparison.

Feeding practices in the semi-solid-feeding group

In the semi-solid group (n = 124), mean scores across FPSQ domains reflected high levels of responsive feeding, as seen in Table 3. The domains family meal environment (M = 3.91; SD = 0.75) and feeding on demand (M = 3.90; SD = 0.78) had the highest mean values. 

**Table 3:** Mean Scores of Feeding Practice in the Semi-solid-Feeding Group P-values represent associations between feeding practice domains and nutritional status assessed using chi-square analysis. Due to sparse cells and the presence of a single Grade 2 case, findings should be interpreted cautiously

Feeding practices	Mean scores	Most common response	Interpretation	P-value (feeding practice and malnutrition)
Feeding on demand	3.90	4	Often	0.185
Family meal environment	3.91	4	Often	0.319
Parent-led feeding	3.59	4	Often	0.008
Persuasive feeding	3.65	4	Often	0.670
Using food to calm	2.81	3	Sometimes	0.541
Using non-food reward	2.90	3	Sometimes	0.418

**Figure 3:**
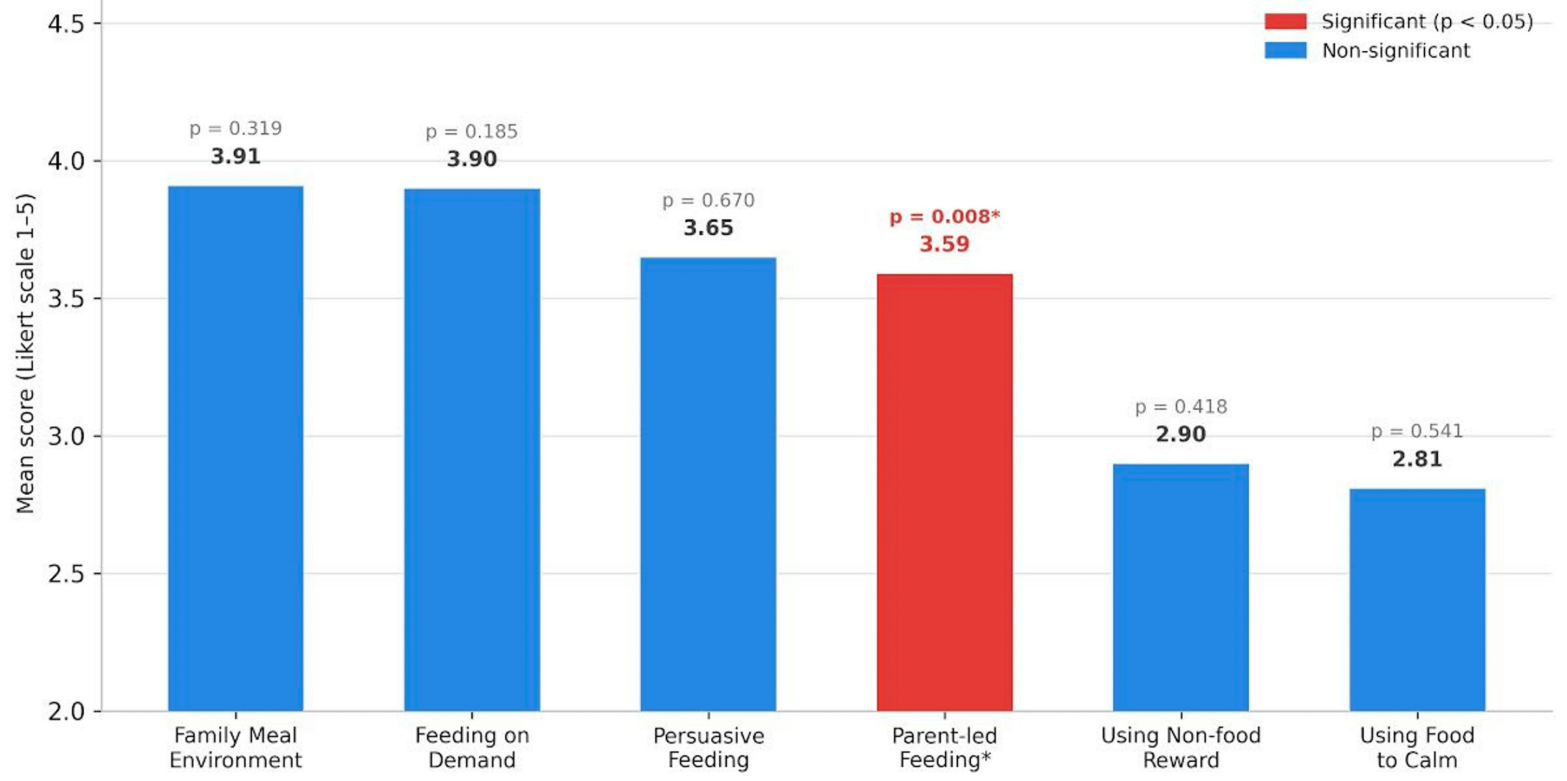
Comparison of Mean Feeding Practice Scores and Their Association With Nutritional Status in Semi-solid-Feeding Group Mean domain scores of the Feeding Practices and Structure Questionnaire (FPSQ) in the semi-solid-feeding group (n = 124). Scores are expressed on a five-point Likert scale (1 = never; 5 = always). Association with nutritional status was assessed using the chi-square test for individual domains. Parent-led feeding was the only domain with a statistically significant association with malnutrition *P < 0.05, statistically significant

Moderate scores were seen for parent-led feeding (M = 3.59; SD = 0.66) and persuasive feeding (M = 3.65; SD = 0.58), suggesting some use of controlling strategies. Lower scores were observed in using food to calm (M = 2.81; SD = 0.88) and non-food rewards (M = 2.90; SD = 0.78) (Figure 3).

Statistical associations between malnutrition and feeding patterns in the semi-solid group

**Figure 4:**
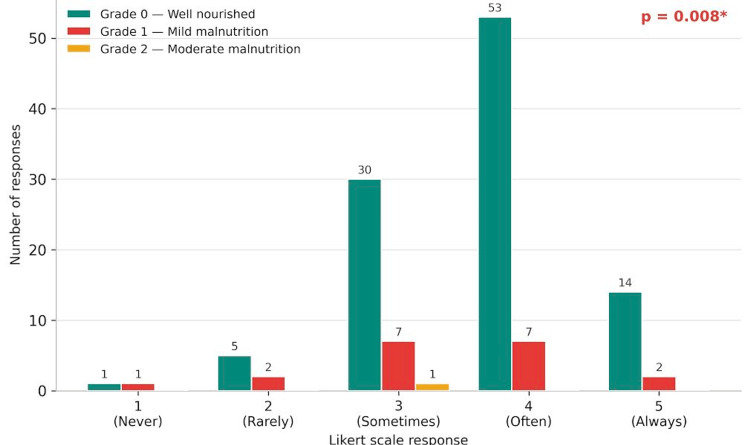
Distribution of Parent-Led Feeding Responses by Nutritional Status Distribution of parent-led feeding responses across Likert scale categories, stratified by nutritional grade, in the semi-solid-feeding group (n = 124). Grade 0 = well-nourished; Grade 1 = mild malnutrition; Grade 2 = moderate malnutrition. Higher parent-led feeding scores were observed among caregivers of malnourished children. Statistical test used: chi-square *P < 0.05, statistically significant

As seen in Figure 4, the chi-square analysis of individual domains revealed that parent-led feeding was associated with malnutrition (p = 0.008), with caregivers of malnourished children reporting higher parent-led feeding scores. Feeding on demand (p = 0.185), family meal environment (p = 0.319), persuasive feeding (p = 0.670), using food to calm (p = 0.541), and non-food rewards (p = 0.418) did not show statistically significant associations. It is acknowledged that the presence of a single Grade 2 case may have contributed to cells with low expected counts in the chi-square analysis, and these findings should therefore be interpreted with caution.

## Discussion

Sociodemographic profile of caregivers and children

The mean age of caregivers in this study was 29.93 years, which falls within the expected range of the appropriate age group for caregiving responsibilities and is consistent with the results of George et al., who noted similar age distribution in caregivers [15]. The vast majority of caregivers were mothers (99.3%), which is reflective of the prevailing cultural norm in India where mothers serve as the primary caregivers and feeding persons for young children, a pattern similarly observed by Pal and Pal [16].

A large proportion of caregivers were graduates, indicating a relatively well-educated sample. Despite this, malnutrition was still observed in some children, suggesting that general educational attainment alone does not necessarily translate into better feeding practices or improved nutritional outcomes. This is consistent with broader evidence that specific nutritional knowledge may play a more targeted role in influencing particular aspects of child growth. For instance, Forh et al. found no significant association between maternal nutritional knowledge and overall malnutrition grades, though a significant association was observed with wasting specifically [9]. This highlights the importance of targeted nutritional counseling that goes beyond general education, with a focus on practical feeding behaviors and responsive caregiving strategies. Socioeconomic status is another important determinant, with lower socioeconomic backgrounds being associated with higher rates of undernutrition and reduced dietary diversity in children across India [16]. The majority of participants in this study belonged to the upper middle class, which may have contributed to the overall favorable nutritional profile of the sample.

The children ranged from one month to five years of age, with a mean of 1.85 years, covering a period of critical dietary transitions from exclusive milk feeding to complementary and solid foods, during which children are particularly vulnerable to nutritional deficiencies [17]. First-born children made up the majority of the sample (56%), and birth order has been shown to influence caregiver feeding behavior, with first-born children often receiving more attentive and intensive feeding care [16]. The mean weight was 10.15 kg (±3.27 kg), and the mean height was 80.2 cm (±14.1 cm), with the wide standard deviations reflecting the broad age range of children included in the study.

Degree of malnutrition

The majority (83.3%) of children were classified as normally nourished, with a smaller proportion experiencing mild (16%) or moderate (0.7%) malnutrition. The low prevalence of severe malnutrition reflects the overall health and resource access of the study population. Comparable findings were reported by Goyal et al., who observed similar rates of undernutrition among under-five children in urban India [17].

Version of the questionnaire

Two tailored versions of the Feeding Practices and Structure Questionnaire (FPSQ) were utilized based on the child's dietary phase: one for milk-fed and one for semi-solid-fed children. The FPSQ framework facilitated the categorization of practices into responsive and controlling types. The FPSQ is a validated tool for assessing caregiver feeding strategies, as confirmed by Jansen et al., who established strong structural reliability in versions for infants and toddlers [12].

Feeding patterns in the milk-feeding group

Among milk-fed children, feeding on demand and using food to calm emerged as the most common practices. These findings reflect a responsive caregiving approach in infancy. Moderate levels of parent-led and persuasive feeding were also reported, indicating occasional reliance on controlling strategies when feeding challenges arise. Costa and Oliveira emphasize that such practices have been associated with child self-regulation and reduce the risk of both under- and overnutrition [4].

Feeding practices and their association with malnutrition in the milk-feeding group

Feeding on demand demonstrated a significant association with better nutritional outcomes. Fildes et al. reported similar benefits, noting that infant appetite patterns and feeding responsiveness have been associated with growth outcomes [6]. These results reinforce the importance of respecting infant hunger and satiety cues, as caregiver responsiveness in early feeding may be associated with adequate energy intake and weight gain.

Feeding patterns in the semi-solid-feeding group

In the semi-solid-feeding group, feeding on demand and inclusion in family meals were the most accepted practices. These behaviors suggest a continued application of responsive feeding as children transition to solids. However, high rates of parent-led and persuasive feeding were also documented, pointing to an underlying concern among caregivers about the child's food intake, as noted by Fontanezi et al. Such controlling behaviors, though well-intentioned, may have been associated with the increased risk of undernutrition or being overweight [10].

An important consideration in interpreting these findings is the possibility of reverse causality. In cross-sectional studies examining feeding practices and nutritional outcomes, it is not possible to determine the direction of association with certainty. Caregivers of children with poor nutritional status may modify their feeding approach in response to perceived underfeeding, for example, increasing parent-led or persuasive feeding when a child is seen to be underweight. This may explain, at least in part, why higher parent-led feeding scores were observed among caregivers of malnourished children in the semi-solid group. Future longitudinal studies are required to establish the temporal direction of these associations.

Strengths and limitations

Strengths

This study utilized the Feeding Practices and Structure Questionnaire (FPSQ), a validated and theory-based tool, enabling the structured assessment of caregiver feeding behaviors. The use of stage-specific FPSQ versions for milk-fed and semi-solid-fed children allowed developmentally appropriate analysis across a heterogeneous age group. Objective anthropometric measurements reduced outcome misclassification. Ethical approval and informed consent were obtained. The study addresses an underexplored area in a tertiary care pediatric outpatient setting in North India, where data on feeding practices using validated tools remain limited.

Limitations

The cross-sectional design precludes causal inference and introduces the possibility of reverse causality, as caregivers may modify feeding practices in response to a child's nutritional status. Being a single-center tertiary care study, the findings are subject to selection bias and limited generalizability. Small subgroup sizes, particularly in the milk-feeding group and the moderate malnutrition category, reduced statistical power. Additionally, several chi-square analyses included cells with low expected counts, affecting the robustness of p-values. Multiple comparisons were conducted without correction, increasing the risk of type I error. No adjustment for confounders was performed.

The questionnaire was administered in participants' preferred language and verbally explained when required; however, formal translation, back translation, and cultural validation were not performed, which may affect measurement validity. Internal consistency (Cronbach's alpha) was not assessed in this sample.

Nutritional status was classified using only weight-for-age (IAP), without assessing wasting or stunting. Effect sizes and confidence intervals were not reported. As feeding practices were self-reported, responses may be influenced by social desirability bias. Additionally, although the dual burden of malnutrition is acknowledged, overweight and obesity were not assessed.

## Conclusions

This exploratory cross-sectional study identified associations between specific caregiver feeding practices and child nutritional status in a tertiary care pediatric outpatient setting. Feeding on demand was associated with better nutritional outcomes in milk-fed children, and parent-led feeding was associated with malnutrition in the semi-solid-feeding group. These findings are associative in nature and, given the cross-sectional design and subgroup limitations, should be interpreted as hypothesis-generating rather than causal. The possibility of reverse causality, whereby caregivers modify feeding practices in response to a child's nutritional status rather than the reverse, cannot be excluded. Longitudinal studies with larger, community-based samples and confounder-adjusted analyses are needed to confirm these associations and establish directionality. Nevertheless, these preliminary findings highlight the potential relevance of responsive feeding counseling in routine pediatric outpatient care as an avenue for nutritional improvement.

## Appendices

Appendix A: Protocol and questionnaire

Table 4 shows the protocol.

**Table 4:** Protocol

Caregiver's details	
Serial number	
Relation of caregiver	
Age	
Gender	
Educational qualification	Illiterate; Grade 10; Grade 12; graduate; postgraduate
Socioeconomic status	
Number of family members	
Child's details	
Unit number	
Name of the child	
Age	
Gender	
Birth order	
Weight (in kg and g)	
Height/length (cm)	
Head circumference (cm)	
Grade of malnutrition	

Table 5 shows the milk-feeding version of the Feeding Pattern and Structure Questionnaire.

**Table 5:** Feeding Pattern and Structure Questionnaire (Milk-Feeding Version) *Reverse-scored items

	Milk-feeding version	Never	Rarely	Sometimes	Often	Always
	Feeding on demand					
1	I feed my baby whenever he wants	1	2	3	4	5
2	I feed my baby at set times*	1	2	3	4	5
3	I decide when it is time for my baby to have a feed	1	2	3	4	5
4	I let my baby decide when he would like to have a feed	1	2	3	4	5
	Parent-led feeding					
5	When deciding how much to feed my baby, I rely on how hungry he/she is*	1	2	3	4	5
6	I feed my baby for a set time	1	2	3	4	5
7	I carefully control how much my baby feeds	1	2	3	4	5
8	I follow a rule about how much my baby should feed	1	2	3	4	5
9	I let my baby decide how much he feeds*	1	2	3	4	5
10	I decide how much my baby feeds	1	2	3	4	5
	Persuasive feeding					
11	I feed my baby extra milk, just to make sure he gets enough	1	2	3	4	5
12	If my baby indicates he is not hungry, I try to get him to feed anyway	1	2	3	4	5
13	I feed my baby extra milk so he sleeps longer	1	2	3	4	5
	Using food to calm					
14	I feed my baby to settle him, even if he is not hungry	1	2	3	4	5
15	I offer my baby a feed when he is unsettled or crying	1	2	3	4	5
16	I offer my baby a feed when he is hurt	1	2	3	4	5
17	When my baby gets unsettled or is crying, feeding him is one of the first things I do	1	2	3	4	5
18	I feed my baby to make sure that he does not get unsettled or cry	1	2	3	4	5

Table 6 shows the semi-solid version of the Feeding Pattern and Structure Questionnaire.

**Table 6:** Feeding Pattern and Structure Questionnaire (Semi-solid Version) *Reverse-scored items

	Semi-solid-feeding version	Never	Rarely	Sometimes	Often	Always
	Feeding on demand					
1	My child eats at set times	1	2	3	4	5
2	I decide when it is time for my child to eat	1	2	3	4	5
3	I let my child decide when she/he would like to eat*	1	2	3	4	5
4	My child has a set mealtime routine	1	2	3	4	5
	Family meal environment					
5	My child eats together with other family members	1	2	3	4	5
6	My child is given the same foods as the rest of the family (pureed, mashed, and chopped)	1	2	3	4	5
7	Whether my child is eating or not, my child sits with the rest of the family when they are having a meal	1	2	3	4	5
8	I eat my meals while my child eats	1	2	3	4	5
	Parent-led feeding					
9	I carefully control how much my child eats	1	2	3	4	5
10	I have a rule about how much my child should eat	1	2	3	4	5
11	I let my child decide how much she/he eats*	1	2	3	4	5
12	I decide how much my child eats	1	2	3	4	5
	Persuasive feeding					
13	I encourage my child to eat all the food in front of him/her	1	2	3	4	5
14	When my child turns away, I try to get her/him to eat a little bit more	1	2	3	4	5
15	If my child indicates she/he is not hungry, I try to get her/him to eat anyway	1	2	3	4	5
16	I say or do something to show my disapproval of my child for not eating	1	2	3	4	5
17	I praise my child after each bit to encourage finishing the food	1	2	3	4	5
18	When my child refuses food they usually eat, I encourage her/him to eat it	1	2	3	4	5
19	I play games to make sure my child eats enough	1	2	3	4	5
	Using food to calm					
20	I give my child food to settle him/her even if he/she is not hungry	1	2	3	4	5
21	I offer my child something to eat to make her/him feel better when she/he is unsettled or crying	1	2	3	4	5
22	I offer my child something to eat to make her/him feel better when she/he is hurt	1	2	3	4	5
23	When my child gets unsettled or is crying, one of the first things I do is give her/him food	1	2	3	4	5
24	I give my child food to make sure that they do not get unsettled or cry	1	2	3	4	5
25	I use food to distract my child or keep him/her busy	1	2	3	4	5
	Using non-food rewards					
26	I offer foods to my child as a reward for good behavior	1	2	3	4	5
27	I offer my child their favorite foods in exchange for good behavior	1	2	3	4	5
28	I promise my child something other than food if they eat (e.g., "If you eat your beans, we can go to the park")	1	2	3	4	5
29	When my child refuses food they usually eat, I encourage eating by offering a non-food reward (e.g., favorite toy or sticker)	1	2	3	4	5
30	I encourage my child to eat something by using food as a reward (e.g., "If you finish your vegetables, you will get some dessert")	1	2	3	4	5
31	When my child refuses food they usually eat, I encourage eating by offering a food reward (e.g., dessert)	1	2	3	4	5
32	I use desserts as an encouragement to get my child to eat the main course	1	2	3	4	5
33	I make my child finish the main course before having a dessert	1	2	3	4	5
34	I warn my child that I will take a favorite food away if my child does not eat a food they do not like (e.g., "If you don't finish your vegetables, you won't get dessert")	1	2	3	4	5

Appendix B: Patient information sheet

This sheet has been prepared to give you all the information needed to make an informed decision about joining this research. Your questions are welcome and will be addressed by the principal investigator or the contacts listed below. After reading through this document and having your questions answered, you may choose to participate. Those who agree will then be asked to sign the consent form.

Research title: Caregiver Feeding Practices and Their Association With Nutritional Status in Children Aged 0-5 Years: A Descriptive Cross-Sectional Study From a Tertiary Care Center in North India

Name and contact details of principal investigator: Sharon Rachel John

1.    What kind of research project is this? Non-funded research project.

2.    Is there any funding involved? No.

3.    What is the purpose of the research project? The purpose of the research is to determine parental perception and its influence on feeding patterns in children aged 0-5 years.

4.    Why have I been chosen to participate in this research project? You have been chosen because your child fits the inclusion criteria of the research.

5.    Do I have to be a part of this research project? Participation in the study is absolutely voluntary. It is completely up to you whether you participate. If you decide not to participate, it will not affect the treatment you receive now or in the future.

6.    What happens if I take part in the research project? The data will be used for the research without revealing the identity.

7.    Will my treatment be withheld if I refuse to take part in this research project? No.

8.    What are the benefits and risks involved in taking part in this research project? This study will help in attaining a better understanding of the caregiver's influence and beliefs in child feeding patterns, which can improve patient care and help in providing better nutritional counseling. There are minimal or no known risks involved as such.

9.    Will the information provided by me be kept confidential? Yes, complete confidentiality will be maintained.

10.  Will my identity be kept anonymous during and after the research project? Yes, the identity of you and your child will be kept confidential.

11.   Will there be any increased cost for participating in this study? None anticipated.

12.   What will be the outcome of the results of this research project? The results of the study will be published without revealing the identity of any participant and with consent.

Please feel free to contact the undersigned if you have any further queries regarding the study:

Sharon Rachel John

In case of clarification, you may contact:

Dr. Jitender Mohindroo

Chairman, Institutional Ethics Committee
